# Anti-infective efficacy of *Psidium guajava* L. leaves against certain pathogenic bacteria

**DOI:** 10.12688/f1000research.17500.2

**Published:** 2019-03-25

**Authors:** Pooja Patel, Chinmayi Joshi, Tannaz Birdi, Vijay Kothari

**Affiliations:** 1 Institute of Science , Nirma University, Ahmedabad, Gujarat, 382481, India; 2 Foundation for Medical Research, Mumbai, India

**Keywords:** Guava leaf, Microwave Assisted Extraction (MAE), Caenorhabditis elegans, Quorum Sensing (QS), Antimicrobial Resistance (AMR), Anti-virulence

## Abstract

Water extracts of *Psidium guajava* leaves prepared by three different extraction methods were compared with respect to their anti-infective activity against *Pseudomonas aeruginosa* and *Staphylococcus aureus* in the nematode host *Caenorhabditis elegans*. The water extract prepared by Microwave Assisted Extraction method was found to have better anti-infective activity, and its activity was further compared with hydroalcoholic extract prepared using the same extraction method against five different pathogenic bacteria. Both these extracts could attenuate virulence of *P. aeruginosa*, *S. aureus*, *Serratia marcescens*, and *Chromobacterium violaceum*, towards *C. elegans. *Anti-infective efficacy of *P. guajava* leaf extract seems partly to stem from its quorum-modulatory property, as it could modulate production of quorum sensing-regulated pigments in all the susceptible bacteria.

## Introduction

Given the heavy global burden of infectious diseases, it is imperative to discover novel pharmaceutical assets for combating antimicrobial resistance, with particular focus on antibiotic-resistant bacterial pathogens recently listed by the World Health Organization as of high/critical priority ( Tacconelli *et al.*, 2018). Since the antibiotic pipeline lacks new mechanisms against resistant bacteria, particularly gram-negative bacteria (see here for more information), it is necessary to look for new antibiotics as well as non-antibiotic approaches to tackle bacterial infections.

A reverse pharmacology approach ( Raut *et al.*, 2017) of investigating plant extracts, particularly those employed in documented or folklore traditional medicine, for their potential anti-pathogenic efficacy may pave the way for discovery and development of novel antimicrobial molecules/ formulations. We undertook the current study to investigate anti-infective potential of one such plant extract, *Psidium guajava* L. (common name- guava; Family- Myrtaceae) leaf extract, against five different pathogenic bacteria. This plant has traditionally been used for treatment of various gastrointestinal problems including diarrhea and dysentery ( Birdi *et al.*, 2010), which are caused usually due to microbial infections. Validation of such traditional medicinal practices through modern scientific approach is necessary for their wider acceptance in the community, and for building public confidence in them ( Kothari, 2018).

## Methods

### Plant material

Shade dried mature guava leaves of *Sardar* variety, one of the five common Indian varieties were used. The leaves were collected in September 2014 from Shirwal, Satara district, Maharashtra, India. The dried leaves were stored in a sealed plastic bag at 25°C. A voucher specimen was deposited at Naoroji Godrej Centre for Plant Research (NGCPR, Shirwal) under herbarium number NGCPR 712.

### Test pathogens

Pathogenic bacteria used in this study ( Dataset 1: Extended data) included *Staphylococcus aureus* (MTCC 737); beta-lactamase producing multidrug resistant strains of *Chromobacterium violaceum* (MTCC 2656) and *Serratia marcescens* (MTCC 97); multidrug resistant *Pseudomonas aeruginosa;* and *Streptococcus pyogenes* (MTCC 1924). Resistance to three or more antibiotics during antibiotic susceptibility profiling ( Dataset 1) was taken as the criteria for tagging any organism as ‘multidrug resistant’. *P. aeruginosa* was sourced from our internal culture collection *.* All other cultures were procured from MTCC (Microbial Type Culture Collection, Chandigarh, India).

### Extraction

In order to identify the best possible extraction method with respect to the desired biological activity, we extracted the powder of the dried leaves in water using three different extraction methods: Decoction, Microwave Assisted Extraction (MAE), and Vacuum Assisted Extraction (VAE). Decoction was selected as one of the methods because this is what the traditional folklore practice has been, whereas MAE and VAE were chosen as additional methods, as they have been known to have the advantages of shorter extraction time, and suitable for extraction of heat-labile phytocompounds too ( Gupta *et al.*, 2012; Wang *et al.*, 2014). Protocols employed for each extraction method are described below:

### Decoction

Decoction of guava leaves was prepared in accordance to the traditional method described in the Ayurvedic texts ( Thakkur, 1976). 1 g of the plant material was boiled in 16 mL double distilled water, till the volume was reduced to 4 mL.

## Microwave Assisted Extraction (MAE) ( Kothari *et al.*, 2009)

1 g of leaf powder was soaked into 16 mL of water or 50% ethanol, and subjected to microwave heating (Electrolux EM30EC90SS) at 720 W. Total extraction duration was 140 s, of which first heating was for 40 s, and subsequent two heating cycles of 10 s each. Intermittent cooling period between any two heating cycles was kept 40 s. Liquid volume at the end of extraction was 4 mL.

## Vacuum Assisted Extraction (VAE)

1 g of dry leaf powder was mixed with 16 mL of water. Vacuum pump (MEDICA INSTRUMENT Mfg. Co.) was attached to the vessel containing plant material and solvent, and the working pressure was set at 7.36 psi (15 In. Hg). Total duration of heating was 20 min, of which for 15 min the system was at 65°C (at which boiling started). Extraction was stopped when liquid volume was reduced to 4 mL.

Extraction performed by methods described above, was followed by macro-filtration using nylon strainer followed by centrifugation (at 10,000 rpm for 15 min; Remi BZCI-8729), and filtration with Whatman paper # 1 (Axiva, Haryana). After this filtration, solvent was evaporated from the extract. For bioassay, extracts was reconstituted in absolute DMSO (Merck, Mumbai). Reconstituted extracts were collected in sterile flat bottom glass vials (15 mL, Merck, Mumbai) covered with aluminum foil, and protected from light to avoid photo-oxidation of light-sensitive compounds. The internal surface of vial cap was also wrapped with aluminum foil to avoid leaching of vial cap material ( Houghton & Raman, 1998). Reconstituted extract was stored under refrigeration for further use. Extraction efficiency was calculated as percentage weight of the starting dried plant material.

Extraction efficiency obtained with these methods was 6.30%, 5.80%, and 6.0% respectively. All the extracts were reconstituted in dimethylsulfoxide (DMSO, Merck) upon drying, and stored under refrigeration (4-8º C) till further use.

*In vivo* efficacy of these water extracts against *Pseudomonas aeruginosa*, and *Staphylococcus aureus* was tested in the nematode host *Caenorhabditis elegans*, wherein the extract prepared by MAE had better anti-infective activity. Therefore, the extract prepared by MAE was compared with its hydroalcoholic extract prepared using the same method. Extraction efficiency obtained for the latter case was 2.0%.

### *In vivo* assay for anti-infective activity

*In vivo* efficacy of the guava leaf extract (GLE) was evaluated using the nematode worm *Caenorhabditis elegans* as the model host, employing the method described by Eng & Nathan, (2015) with some modification. *C. elegans* was maintained on Nematode Growing Medium (NGM) which consisted of 3 g/L NaCl, 2.5 g/L peptone, 1 M CaCl _2_, 1 M MgSO _4_, 5 mg/mL cholesterol, 1 M phosphate buffer of pH 6, 17 g/L agar-agar with *E. coli* OP50 (procured from LabTIE B.V., JR Rosmalen, the Netherlands) as the feed. The worm population to be used for the *in vivo* assay was kept on NGM plates not seeded with *E. coli* OP50 for three days, before being challenged with the test pathogen.

Pathogenic bacteria was incubated with GLE for 22-24h (48 h in case of *S. marcescens* and *S. aureus*) at 37°C (28°C for *S. marcescens*). Appropriate vehicle control was also set wherein GLE was replaced with DMSO (0.5%v/v). Following incubation, OD _764_ of the bacterial culture (grown in presence of GLE) suspension was equalized to that of the DMSO control. 100 μL of this bacterial suspension was mixed with 900 μL of the M9 buffer containing 10 worms (L3-L4 stage). This experiment was performed in 24-well (sterile, non-treated) polystyrene plates (HiMediaTPG24), and incubation was carried out at 22°C. Number of live vs. dead worms was counted daily for five days by putting the plate (with lid) under light microscope (4X). Standard antibiotic (gentamicin)- and catechin- treated bacterial suspension were used as positive control; since gentamicin is a known broad-spectrum bactericidal antibiotic ( Fitzgerald & Newquist, 2013), and catechin is a known anti-infective agent capable of modulating bacterial quorum sensing ( Joshi *et al.*, 2019) Straight worms were considered to be dead, Plates were gently tapped to confirm lack of movement in the dead-looking worms. On the last day of the experiment, when plates could be opened, their death was reconfirmed by touching them with a straight wire, wherein no movement was taken as confirmation of death.

### Statistical analysis

Values reported are means of four independent experiments, whose statistical significance was assessed using *t*-test performed through Microsoft Excel (2013). *P* values ≤0.05 were considered to be statistically significant.

## Results

**Figure 1.**
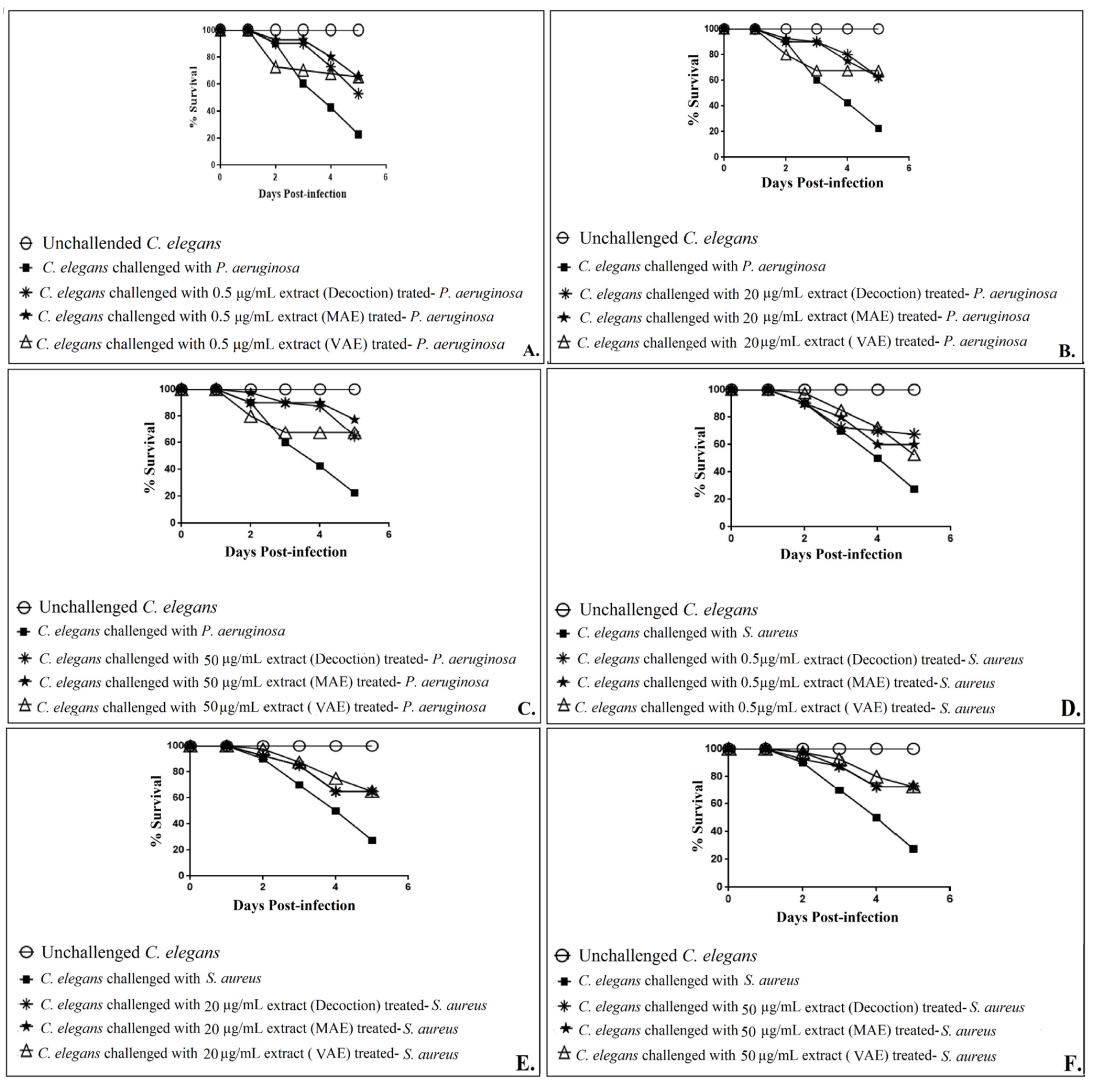
Comparison of *in vivo* anti-infective efficacy of *P. guajava* leaf extracts prepared by three different extraction methods, against *P. aeruginosa* ( **A**– **C**), and *S. aureus* ( **D**– **F**). Catechin (50 μg/mL) and gentamicin (0.1 μg/mL) employed as positive controls conferred 100% and 80% protection on the worm population, respectively. DMSO present in the ‘vehicle control’ at 0.5%v/v did not affect virulence of the bacterium towards *C. elegans*. DMSO (0.5%v/v) and GLE at tested concentrations showed no toxicity towards *C. elegans*. MAE: Microwave Assisted Extraction; VAE: Vacuum Assisted Extraction; GLE: Guava Leaf Extract.

GLE prepared by three different methods were compared, at three different concentrations, for their anti-infective activity against *P. aeruginosa* and *S. aureus* ( Figure 1; Dataset 1: Underlying data). At 50 µg/mL, GLE prepared by MAE proved superior to that prepared by decoction or VAE method, with respect to its ability to attenuate *P. aeruginosa*’s virulence towards *C. elegans*. At 0.5 µg/mL, extract prepared by decoction method registered least activity against this bacterium. At the same concentration, against *S. aureus*, extract prepared by VAE displayed the least activity. Based on these results, we concluded MAE as a better extraction method, and then extracted guava leaves using this method in water as well as water:alcohol (1:1) mixture. Both of these extracts prepared using MAE were then assayed for their anti-infective potential against five different pathogenic bacteria.

**Figure 2.**
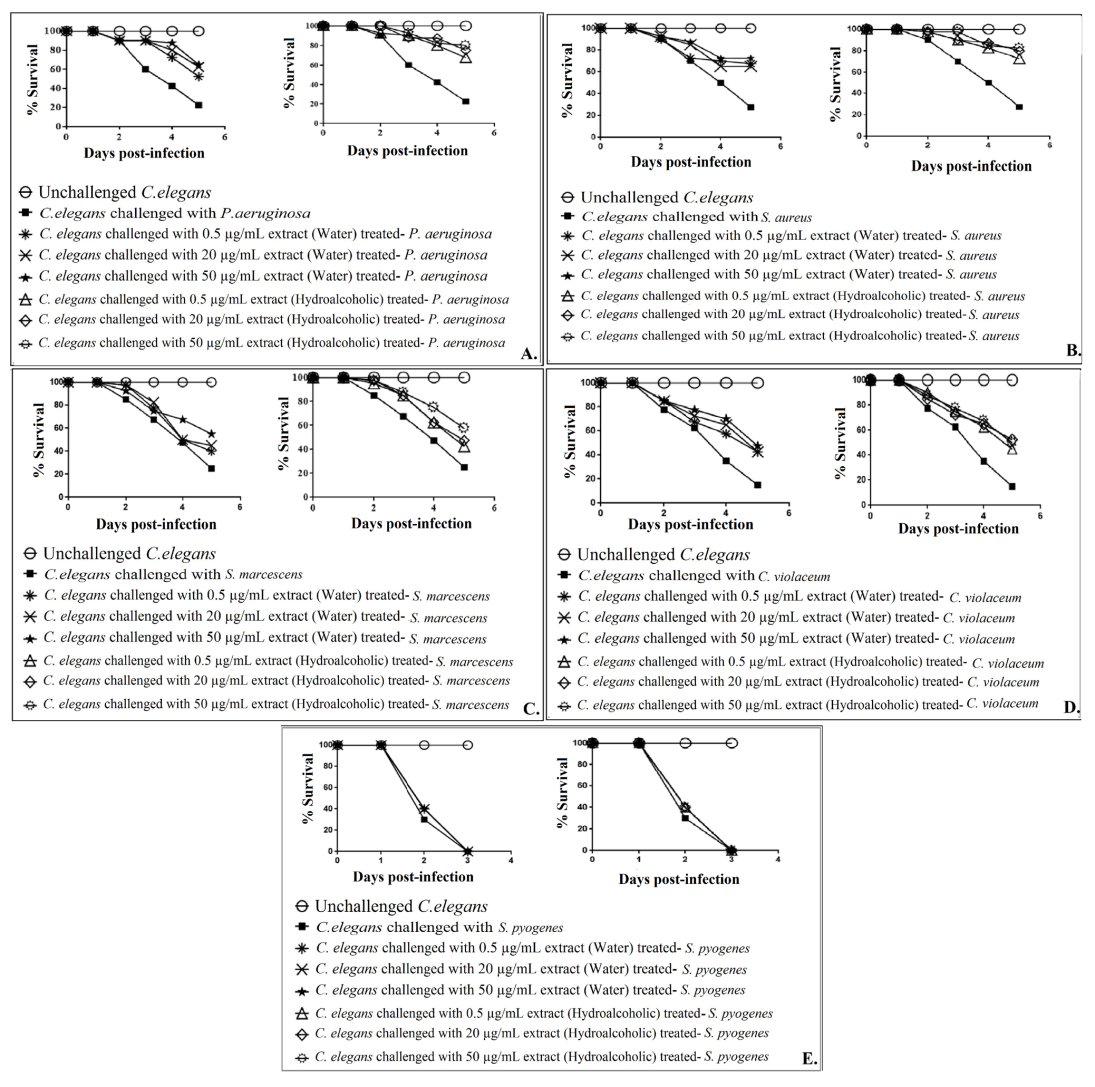
Comparison of the *in vivo* anti-infective potential of water extract and hydroalcoholic extract of *P. guajava* leaf extracts prepared by Microwave Assisted Extraction method, against five different pathogenic bacteria. Figures **A**– **E** shows data against *P. aeruginosa, S. aureus, S. marcescens, C. violaceum* and *S. pyogenes* respectively. Catechin (50 μg/mL) employed as a positive control conferred 100% protection on worm population against all the pathogenic bacteria except *S. pyogenes*. Against *S. pyogenes*, catechin could not offer any protection to host worms. Gentamicin (0.1 μg/mL) allowed survival of worm population to the extent of 80% in face of *P. aeruginosa*, *S. aureus*, or *S. pyogenes* challenge; and 100% against the remaining two pathogens. DMSO present in the ‘vehicle control’ at 0.5%v/v did not affect virulence of the bacteria towards *C. elegans*. DMSO (0.5%v/v) and GLE at tested concentrations showed no toxicity towards *C. elegans*.

Both water as well as the hydroalcoholic extract of guava leaves could attenuate virulence of all the test pathogens (except *S. pyogenes*) towards *C. elegans* ( Figure 2; Dataset 1: Underlying data). Both these extracts exhibited statistically similar anti-pathogenic efficacy against all susceptible bacteria, but the hydroalcoholic extract exhibited 10-15% better activity against *S. aureus* than the water extract. Despite the lowest extraction yield among all extracts reported in this study, the hydroalcoholic GLE was found to possess the highest (at par with water extract against all gram-negative pathogens) anti-pathogenic activity. Critical importance of choice of most appropriate extraction method and solvent for preparation of bioactive extracts has earlier been also emphasized by us ( Gupta *et al.*, 2012; Kothari *et al.*, 2012), and others ( Ngo *et al.*, 2017; Sasidharan *et al.*, 2011).

**Figure 3.**
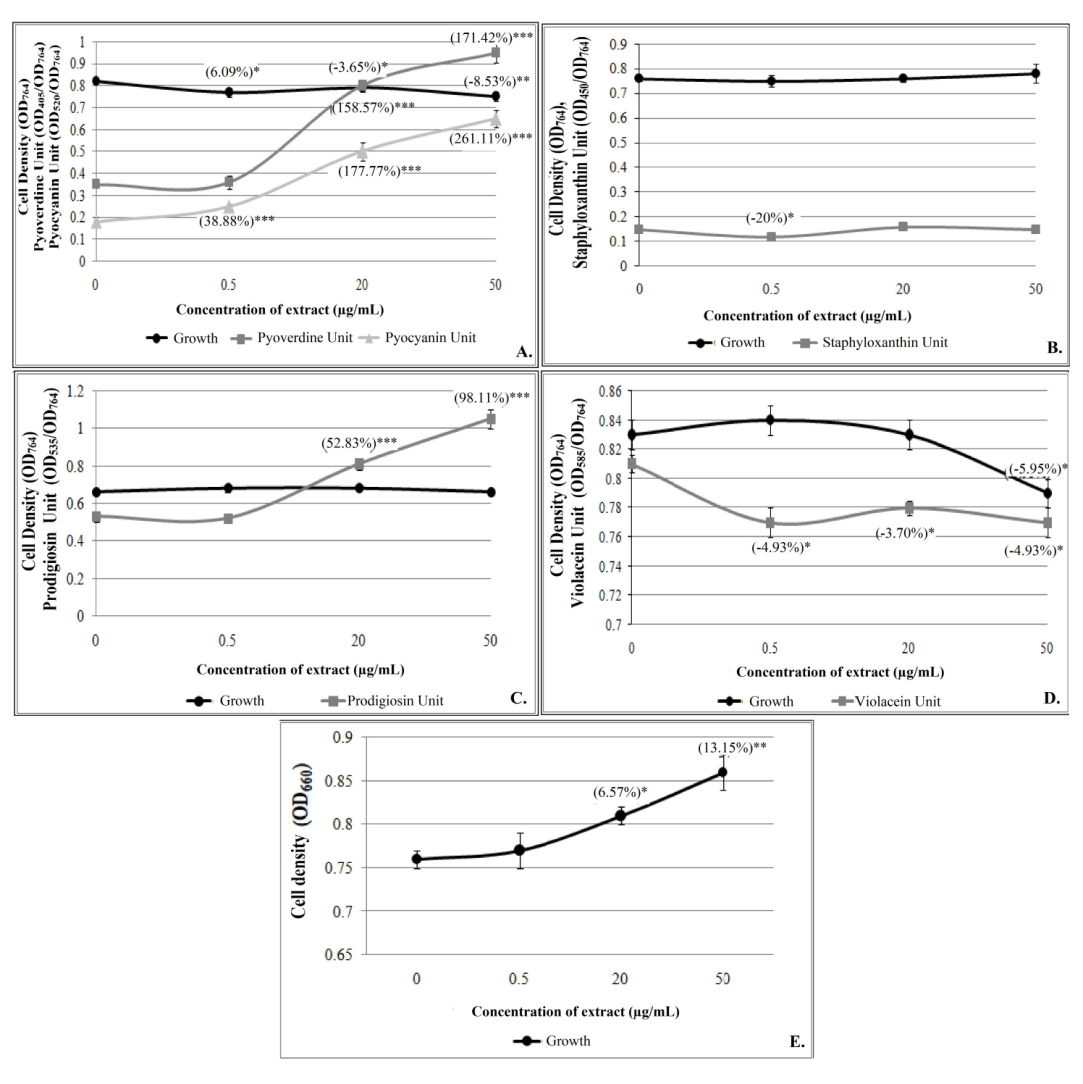
Effect of hydroalcoholic extract of *P. guajava* leaves prepared by Microwave Assisted Extraction method on bacterial growth and QS-regulated pigment production. ( **A**) *P. aeruginosa* ( **B**) *S. aureus* ( **C**) *S. marcescens* ( **D**) *C. violaceum* ( **E**) *S. pyogenes.* Bacterial cell density and pigment production were quantified as earlier described by us ( Joshi *et al.*, 2016). Bacterial growth was measured as OD _764_ for the four pigmented bacteria, while for *S. pyogenes* OD _660_ was used. OD of pyoverdine was measured at 405 nm, and that of pyocyanin at 520 nm; Pyoverdine Unit was calculated as the ratio OD _405_/OD _764_ (an indication of pyoverdine production per unit of growth); Pyocyanin Unit was calculated as the ratio OD _520_/OD _764_ (an indication of pyocyanin production per unit of growth. OD of staphyloxanthin was measured at 450 nm, and Staphyloxanthin Unit was calculated as the ratio OD _450_/OD _764_ (an indication of staphyloxanthin production per unit of growth). OD of prodigiosin was measured at 535 nm, and Prodigiosin Unit was calculated as the ratio OD _535_/OD _764_ (an indication of prodigiosin production per unit of growth). OD of violacein was measured at 585 nm, and Violacein Unit was calculated as the ratio OD _585_/OD _764_ (an indication of violacein production per unit of growth). QS: Quorum sensing

To have some insight into the mode of action of GLE, we incubated all the five test bacteria with GLE to investigate whether it affects bacterial growth and/or quorum-sensing (QS) regulated pigment production (a marker trait). Bacterial cell density and pigment production were quantified as earlier described by us ( Joshi *et al.*, 2016; Patel *et al.*, 2018). Pigment production in all the four pigmented bacteria was modulated at ≥1 concentration(s) of the GLE tested. ( Figure 3; Dataset 1: Underlying data). Since this extract did not inhibit bacterial growth heavily, it can be expected to exert lesser selection pressure (as opposed to potent bactericidal agents) on susceptible bacterial populations, and may not induce rapid development of resistant phenotypes. Ability of GLE to interfere with bacterial QS is an important observation, as QS in recent years has emerged as a potential target for novel anti-pathogenic agents ( Fong *et al.*, 2018). These ‘pathoblockers’ may attenuate virulence of the target pathogens without necessarily killing them ( Kamal *et al.*, 2017).

Details of organisms used in this study including antibiogram Click here for additional data file. Copyright: © 2019 Patel P et al. 2019 Data associated with the article are available under the terms of the Creative Commons Zero "No rights reserved" data waiver (CC0 1.0 Public domain dedication).

Raw data for Figures 1-3 showing the anti-infective efficacy of *Psidium guajava* L. leaves against pathogenic bacteria Click here for additional data file. Copyright: © 2019 Patel P et al. 2019 Data associated with the article are available under the terms of the Creative Commons Zero "No rights reserved" data waiver (CC0 1.0 Public domain dedication).

## Conclusion

 Results of the present study validate the traditional use of guava leaves for medicinal purposes and suggests one of the possible mechanisms through which it exerts its anti-infective activity, i.e. its ability to interfere with the bacterial QS machinery. Further investigation regarding GLE’s effect on pathogenic bacteria at the whole transcriptome level is warranted to unravel the molecular mechanisms underlying its anti-pathogenic efficacy.

## Data availability

The data referenced by this article are under copyright with the following copyright statement: Copyright: © 2019 Patel P et al.

Data associated with the article are available under the terms of the Creative Commons Zero "No rights reserved" data waiver (CC0 1.0 Public domain dedication). 

### Underlying data

F1000Research: Raw data for Figure 1– Figure 3 showing the anti-infective efficacy of Psidium guajava L. leaves against pathogenic bacteria., https://doi.org/10.5256/f1000research.17500.d230522 ( Patel *et al.*, 2018a).

### Extended data

F1000Research: Details of organisms used in this study including antibiogram., https://doi.org/10.5256/f1000research.17500.d230521 ( Patel *et al*., 2018b).
