# Supplementary material for: Anti-infective efficacy of Psidium guajava L. leaves against certain pathogenic bacteria
Source: F1000Res. 2019 Mar 25;8:12. Originally published 2019 Jan 3. [Version 2] doi: 10.12688/f1000research.17500.2 (PMC6468707; doi:10.12688/f1000research.17500.2)
Supplement: Details of organisms used in this study including antibiogram [file f1000research-8-20309-s0000.tgz › a99f9a84-6323-409f-9cfc-eab4f32699d0_Organisms_used_in_the_study.docx]

| **Organism** | **Source** | **Growth medium** | **Incubation time** | **Incubation temperature** | **QS-regulated pigment of the strain** |
| --- | --- | --- | --- | --- | --- |
| ***C. violaceum***  **(MTCC 2656)** | Microbial Type Culture Collection (MTCC), Chandigarh | Nutrient broth  (HiMedia, Mumbai) | 22-24 h | 37 ^˚^C | Violacein |
|  |  |  |  |  |  |
| ***P. aeruginosa*** | Our institutional culture collection | Pseudomonas broth |  |  | Pyoverdine  Pyocyanin |
| ***S. aureus***  **(MTCC 737)** | MTCC | Standard staphylococcus broth with 0.5% yeast extract | 46-48 h | 37 ˚C | Staphyloxanthin |
| ***S. marcescens* (MTCC 97)** | MTCC | Nutrient broth  (HiMedia, Mumbai) |  | 28˚C | Prodigiosin |
| ***S. pyogenes***  **(MTCC 1924)** | MTCC | Brain Heart Infusion broth (HiMedia, Mumbai) | 22-24 h | 37 ˚C | Non-pigmented strain |
|  |  |  |  |  |  |

**Details of organisms used in this study**

**Antibiotic susceptibility profile**

| **Antibiotic** | **Concentration (µg)** | **Result** | | |
| --- | --- | --- | --- | --- |
|  |  | ***C. violaceum*** | ***S. marcescens*** | ***P. aeruginosa*** |
| Amikacin | 30 | S | S | S |
| Norfloxacin | 10 | S | S | I |
| Erythromycin | 15 | S | I | R |
| Amoxycillin/ Clavulanic acid | 10 | I | I | R |
| Co- trimoxazole | 25 | S | S | R |
| Ciprofloxacin | 5 | S | S | S |
| Netillin | 10 | S | S | S |
| Gentamicin | 10 | S | S | I |
| Cefadroxil | 30 | R | R | R |
| Ampicillin | 10 | R | R | R |
| Penicillin | 10 units | R | R | R |
| Tobramycin | 10 | S | S | R |
| Nalidixic acid | 10 | S | S | S |
| Ceftazidime | 30 | S | S | I |
| Cephoperazone | 75 | S | S | I |
| Vancomycin | 30 | I | R | R |
| Chloramphenicol | 30 | S | S | R |
| Ceftriaxone | 30 | S | S | R |
| Nitrofurantion | 10 | S | R | R |
| Cloxacillin | 1 | R | R | R |
|  |  | ***S. aureus*** | | |
| Penicillin G | 10 units | S | | |
| Oxacillin | 1 | S | | |
| Erythromycin | 15 | S | | |
| Clindamycin | 2 | S | | |
| Linezoild | 30 | S | | |
| Co- Trimoxazole | 25 | S | | |
| Vancomycin | 30 | S | | |
| Ciprofloxacin | 5 | S | | |
| Tetracycline | 30 | S | | |
| Cefotaxime | 30 | S | | |
| Chloramphenicol | 30 | S | | |
| Gentamicin | 10 | S | | |

Antimicrobial susceptibility was interpreted from zones of inhibition (diameter in mm); S: susceptible (diameter of zone of inhibition 16 mm or more) ; R: resistant (diameter of zone of inhibition 10 mm or less; I: Intermediate (diameter of zone of inhibition 11 mm-15 mm ) [Dodeca Universal–I, Dodeca G-III–Plus, and Icosa Universal-2 (HiMedia, Mumbai)]
