# Supplementary material for: Anti-infective efficacy of Psidium guajava L. leaves against certain pathogenic bacteria
Source: F1000Res. 2019 Mar 25;8:12. Originally published 2019 Jan 3. [Version 2] doi: 10.12688/f1000research.17500.2 (PMC6468707; doi:10.12688/f1000research.17500.2)
Supplement: Peer review history

# Peer review: Reviewer response for version 2

**DOI:** 10.5256/f1000research.20309.r46154

**Published:** 2019-04-15

**Authors:** Virupakshi Soppina

**Affiliations:** 1 Indian Institute of Technology Gandhinagar, Gandhinagar, Gujarat, India

Authors have satisfactorily addressed the concerned raised. I have no further comments to make.

I have read this submission. I believe that I have an appropriate level of expertise to confirm that it is of an acceptable scientific standard.

# Peer review: Reviewer response for version 1

**DOI:** 10.5256/f1000research.19139.r42508

**Published:** 2019-02-25

**Authors:** Vivekananda Mandal

**Affiliations:** 1 Institute of Pharmacy, Guru Ghasidas Central University, Bilaspur, Chhattisgarh, India

In this short study, the authors have studied anti-pathogenic potential of *P. gujava* leaves, which is an important plant in traditional medicine. It is good to see that among test bacteria, authors have included multi-drug resistant/beta-lactamase producing gram-negative bacteria, as it is difficult to find 'hits' against gram-negative bacteria in general. Their idea of comparing the same leaf extract prepared using different extraction methods also seems to be logical, as choice of the most appropriate extraction method is very much crucial while assessing the biological activity of plant extracts. It can have a significant bearing on the final results.

 They have found MAE to be a good method. MAE has earlier been also reported by various groups to be an efficient extraction method, particularly for fast extraction of plant phenolic compounds. Further, they have used the worm *C. elegans* as the model host for their test pathogens. This worm is a good choice for generating useful preliminary data on *in vivo* efficacy of potential anti-pathogenic extracts/ formulations.

 In the case of some bacteria like *P. aeruginosa*, there is an overlap among virulence factors (e.g. pyocyanin) responsible for damaging the human cells and those killing the worm. They have also compared the GLE prepared in water vs. that prepared in water + alcohol, and have emphasized the importance of choice of most appropriate extraction method and solvent for preparation of bioactive extracts.

 Their *in vitro* experiments have provided a good clue on one of the possible ways regarding mode of action of GLE i.e. QS interference. QS in recent years has been reported by many research groups to be a target worth pursuing, in search of novel antimicrobials. Raw data submitted by the authors also seem to be in good shape, and in line with their findings reported in main text.

 Overall, this seems to be an okay study, and can be approved for indexing without any major changes. However, in future the authors should try to come up with a full-length report describing molecular mechanisms at the genome/transcriptome level explaining the mechanistic basis of GLE's anti-pathogenic efficacy.

I have read this submission. I believe that I have an appropriate level of expertise to confirm that it is of an acceptable scientific standard.

# Peer review: Reviewer response for version 1

**DOI:** 10.5256/f1000research.19139.r42519

**Published:** 2019-01-21

**Authors:** Virupakshi Soppina

**Affiliations:** 1 Indian Institute of Technology Gandhinagar, Gandhinagar, Gujarat, India

Patel et al. study the anti-infective properties of *Psidium guajava *leaf extract, against five different pathogenic bacteria. They use *C. elegans* as a model system to study the anti-infective efficiency of *P. guajava *leaf extract formulated from three different extraction methods. Overall this is an exciting paper, validates the traditional use of guava leaves for medicinal purposes and also a possible mechanism. The topic is important, and this paper adds something new to the pharmacology field.  

 The key finding of this study is that the water and hydroalcoholic extracts prepared using microwave-assisted extraction method could successfully attenuate the virulence of different pathogenic bacteria and also exhibit anti-infective property towards *C. elegans*. I do not have any significant concerns or comments on the manuscript. However, there are some minor comments to improve the manuscript readability and understand the experiments.

1. The manuscript needs a more relevant background to understand the significance of the manuscript.
2. It would be useful to state why the authors have specifically used three extraction methods that are used in the paper over several extraction methods available in the field.
3. Under *In vivo* assay for anti-infective activity section, the sentence ‘ Pathogenic bacteria were incubated with GLE for 22-24h (48h in case of *S. marcescens* and *S. aureus*) at 37°C (28°C for *S. marcescens*). Following incubation, OD764 of the culture suspension was equalized to that of the DMSO control.’ is highly confusing so please rewrite with precise details.
4. The sentence ‘ *Number of live vs. lead worms was counted daily for five days by putting the plate*’ should be written as ‘ *Number of live vs. dead worms was counted daily for five days by putting the plate*.’
5. In Figure 1B, please use consistent symbol shapes for each data set.
6. Graphs in Figure 2 are too small and crowded (it is difficult to appreciate the results), so please consider increasing the size of graphs or using different symbol shapes or think of presenting the data in bar graph format.
7. Please include the data for positive controls [catechin (50 μg/mL) and gentamicin (0.1 μg/mL)] in Figure 1 and 2.
8. Please provide scientific background for using catechin and gentamicin as positive controls.
9. The sentence ‘ At least one concentration of GLE was found to modulate pigment production in all the four pigmented bacteria (Figure 3; Dataset 1: Underlying data). This extract did not inhibit bacterial growth heavily, and hence can be expected to exert lesser selection pressure on susceptible bacterial populations.’ is difficult to understand so please consider rewriting with clear statements.
10. The results of Figure 3 need more discussion in further details in the context of published literature.

I have read this submission. I believe that I have an appropriate level of expertise to confirm that it is of an acceptable scientific standard, however I have significant reservations, as outlined above.

# Response

**Published:** 2019-03-16

**Authors:** Vijay Kothari

**Affiliations:** Nirma university, India

We thank both the referees for devoting their time in reviewing our manuscript. Our comment-wise response to referee-1’s comments is as under:

- Comment 1: A line has been added in ‘Introduction’ telling the significance of such studies aimed at validating the traditional medicine claims.
- Comment 2: Basis of selection of these three extraction method has been added in the ‘Methods’ section under subheading ‘Extraction’.
- Comments 3,4, and 9: Sentences have been rewritten to correct spelling mistake, and add clarity.
- Comment 5: Error regarding symbol shape has been corrected in the revised version of Figure-1.
- Comment 6: To avoid the crowded appearance of Figure-2, in the revised version, we have divided all the five parts A-E into two separate graphs, one for water extract, and another for hydroalcoholic extract.
- Comment 7: Data for positive controls has already been there in legends of Figure 1-2. Adding separate lines for them in graph will again make the figures crowded.
- Comment 8: Scientific background for selection of positive controls has been added under the heading “ *In vivo* assay for anti-infective activity”.
- Comment 10: Relevant content has been added discussing the results of Figure-3, citing appropriate references.

Since this is a short ‘Research Note’, we have focused more on presenting our results, and refrained from adding too much content for ‘Discussion’.
